# c-Jun N-terminal kinases differentially regulate TNF- and TLRs-mediated necroptosis through their kinase-dependent and -independent activities

**DOI:** 10.1038/s41419-018-1189-2

**Published:** 2018-11-15

**Authors:** Mengtao Cao, Fei Chen, Ni Xie, Meng-Yao Cao, Pengfei Chen, Qi Lou, Yanli Zhao, Chen He, Shuyuan Zhang, Xinyang Song, Yu Sun, Weimin Zhu, Lisha Mou, Shaodong Luan, Hanchao Gao

**Affiliations:** 1 Institute of Transformation Medicine, Shenzhen Second People’s Hospital, First Affiliated Hospital of Shenzhen University, Shenzhen University School of Medicine, 518300 Shenzhen, China; 2 The Key Laboratory of Stem Cell Biology, Institute of Health Sciences, Shanghai Institutes for Biological Sciences, Chinese Academy of Sciences, University of Chinese Academy of Science, 200031 Shanghai, China; 3 Department of Radiology, The People’s Hospital of Tongliang District, 402560 Chongqing, China; 4 Department of Ophthalmology, Shenzhen Second People’s Hospital, First Affiliated Hospital of Shenzhen University, 518035 Shenzhen, Guangdong China; 5 Department of Nephrology, Shenzhen Longhua District Central Hospital, Guangdong Medical University Affiliated Longhua District Central Hospital, 518300 Shenzhen, China; 6 Division of Immunology, Department of Microbiology and Immunobiology, Harvard Medical School, Boston, MA 02115 USA; 7 Department of Sports Medicine, Shenzhen Second People’s Hospital, First Affiliated Hospital of Shenzhen University, 518035 Shenzhen, Guangdong China

## Abstract

Tumor necrosis factor (TNF) and Toll-like receptor (TLR)3/TLR4 activation trigger necroptotic cell death through downstream signaling complex containing receptor-interacting protein kinase 1 (RIPK1), RIPK3, and pseudokinase mixed lineage kinase-domain-like (MLKL). However, the regulation of necroptotic signaling pathway is far less investigated. Here we showed that c-Jun N-terminal kinases (JNK1 and JNK2) displayed kinase-dependent and -independent functions in regulating TNF- and TLRs-mediated necroptosis. We found that RIPK1 and RIPK3 promoted cell-death-independent JNK activation in macrophages, which contributed to pro-inflammatory cytokines production. Meanwhile, blocking the kinase activity of JNK dramatically reduced TNF and TLRs-induced necroptotic cell death. Consistently, inhibition of JNK activity protected mice from TNF-induced death and *Staphylococcus aureus*-mediated lung damage. However, depletion of JNK protein using siRNA sensitized macrophages to necroptosis that was triggered by LPS or poly I:C but still inhibited TNF-induced necroptosis. Mechanistic studies revealed that RIPK1 recruited JNK to the necrosome complex and their kinase activity was required for necrosome formation and the phosphorylation of MLKL in TNF- and TLRs-induced necroptosis. Loss of JNK protein consistently suppressed the phosphorylation of MLKL and necrosome formation in TNF-triggered necroptosis, but differentially promoted the phosphorylation of MLKL and necrosome formation in poly I:C-triggered necroptosis by promoting the oligomeration of TRIF. In conclusion, our findings define a differential role for JNK in regulating TNF- and TLRs-mediated necroptosis by their kinase or scaffolding activities.

## Introduction

Necroptosis is a type of programmed cell death, independent of caspases activity, and characterized by membrane swell and rupture, which is mediated by RIPK1 and RIPK3^1^. Various stimuli can trigger necroptosis, including activation of specific death receptors, interferon receptors, or TLRs^2–6^. Upon the stimulation of TNF, RIPK1 and RIPK3 form a protein complex called necrosome through their RIP homotypic interaction motif (RHIM), which leads to the phosphorylation and oligomerization of RIPK3^7–9^. Activated RIPK3 subsequently phosphorylates the downstream pseudokinase MLKL^10,11^. Ultimately, phosphorylated MLKL undergoes oligomerization and translocates to the cell membrane to execute necroptosis^12,13^. Excessive and uncontrolled necroptosis lead to embryonic development failure. Also, under many pathophysiology settings, including viral or bacterial infection, pancreatitis, multiple sclerosis, neurodegeneration, and systemic inflammatory response syndrome, the significance of necroptosis has been revealed^5,6,8,14–17^.

Necroptotic signaling pathway has been reported to be tightly regulated, especially in the embryonic development^1,18^. Several apoptosis-related proteins, including caspase 8, FADD, and cFLIP, inhibit the activation of necroptosis. Deficiency of these genes causes embryonic lethality in mice which is dependent on RIPK3 and MLKL-mediated necroptosis^19–21^. Other reports showed that several kinases, including IKKα, IKKβ, and MK2 directly phosphorylated RIPK1 and restricted the autophosphorylation of RIPK1, which inhibited the activation of TNF-induced apoptosis and necroptosis^22–25^. Recently, it has been reported that ppm1b dephosphorylated RIPK3 and CHIP mediated the ubiquitination and lysosome-dependent degradation of RIPK3^26,27^. Besides the negative regulation of necroptotic signaling pathway, some genes positively regulated necroptotic signaling pathway. Cyld can deubiquitylate RIPK1 and facilitate TNF-induced necroptosis, which can be cleaved by caspase 8^28,29^. Additionally, necrosome recruits molecular chaperone HSP90 and CDC37 to facilitate necroptotic signaling transduction^30^.

c-Jun N-terminal kinases or JNKs are a subfamily of the mitogen-activated protein kinase superfamily, which contains three isoforms (JNK1, JNK2, and JNK3) with multiple splice variants^31^. JNK1 and JNK2 are ubiquitously expressed, while JNK3 mainly exists in neuronal and heart tissues^32^. JNKs have been reported to be involved in the regulation of multiple cellular processes, from apoptosis to gene expression^33^. JNKs show a critical role in death receptors induced by extrinsic and mitochondrial intrinsic apoptotic pathways. Other studies also have showed that JNK function as a double-edged sword in the regulation of apoptosis and had pro- or antiapoptotic functions, dependent on different cell types and death stimulus^32^. Although JNK has been reported to regulate apoptosis, the functional roles of JNK in necroptosis have not been well characterized. Here we report that JNK differentially regulate TNF- and TLRs-mediated necroptosis by their kinase and scaffolding activities.

## Materials and methods

### Experimental reagents

The following compounds were used: Necrostatin-1 (50μM, Calbiochem), SP600125 (10μM, Selleck), SB203580 (10μM, Selleck), PD98059 (10μM, Selleck), zVAD (20μM, ENZO), Smac (SM-164, 100 nM, MedchemExpress), butylated hydroxyanisole (BHA) (100μM, Sangon Biotec), and protonophore carbonylcyanide m-chlorophenylhydrazone (CCCP) (12.5μM, Sigma). LPS and poly I:C were purchased from InvivoGen. Mouse recombinant TNF was purchased from Ebioscience. Propidium iodide (PI) was from Sigma.

### Cells

MEF cells, L929 cells, and Raw 264.7 cells were cultured in complete Dulbecco's modified Eagle's medium supplemented with 10% FBS, 100U/ml penicillin, and 100μg/ml streptomycin.

Bone marrow-derived macrophages (BMDMs) were prepared from bone marrow collected from the femurs and tibias of wild-type mice. Cells were depleted red cells with ammonium chloride and were differentiated over 6 days in the presence of conditional media (DMEM containing 10% FBS, 100U/ml penicillin, 100 μg/ml streptomycin, and 30% L929 conditioned medium). Fresh media were changed every 2 days. After 6 days, adherent cells were collected and reseeded for further experiments.

Peritoneal macrophages (PMs) were isolated and cultured as described previously^34^. Briefly, wild-type mice or TNF-deficient mice were injected intraperitoneally (i.p.) with 2ml of 4% thioglycolate medium (FTG from BD Biosciences). After 3 days, cells were collected by peritoneal cavity lavage with sterile PBS and seeded in the plates in DMEM complete media. After 2 h, the supernatant cells were discarded and the adherent cells were regarded as peritoneal macrophages.

### Mice

Wild-type C57BL/6 mice were purchased from Slac Laboratory Animal Center, Chinese Academy of Sciences. TNF-deficient mice (strain B6.129S-Tnf^tm1Gkl/J^) were from Model Animal Research Center of Nanjing University. All mice were maintained in specific pathogen-free conditions at the Institute of Health Sciences. Six to ten weeks old and gender-matched mice were used in all experiments. All animal experiments were performed according to the guidelines for the care and use of laboratory animals and are approved by the institutional biomedical research ethics committee of the Shanghai Institutes for Biological Sciences (Chinese Academy of Sciences).

### RNA interference

Raw 264.7 cells, MEF cells, and peritoneal macrophages were seeded in 96-well plates to detect cell viability or 12-well plates to harvest protein or stain with propidium iodide (PI). Cells were transiently transfected with scrambled siRNA (negative control), RIPK3 siRNA (5′-gcucucgucuucaacaacu-3′), MLKL siRNA (5′-gaaccugcccgaugacauu-3′), JNK oligo (JNK1 siRNA 5′-ugauucagauggaguuaga-3′, JNK2 siRNA 5′-ccgcagaguucaugaagaa-3′), JNK-oligo-2 siRNA (JNK1 siRNA 5′-aauauagucccuuccuuggaaagagg-3′, JNK2 siRNA 5′-uucaaucgcaugcucucuuucuucc-3′), and RIPK1 siRNA (5′-gcauuguccuuugggcaauau-3′). siRNAs were transfected using Lipofectamine RNAiMAX (Life Technologies), according to the manufacturer’s protocol. The cells were incubated with the transfectin mixture for 3 days, and then treated with indicated ligands and inhibitors. Knock down efficiency was determined by detecting protein levels or mRNA levels.

### Real-time PCR

Peritoneal macrophages were seeded in 12-well plates and then treated as indicated. RNA of peritoneal macrophages was prepared using the TRIzol reagent according to the manufacturer’s protocol. cDNA was produced by retro-transcribing RNA using PrimeScript RT reagent kit (Takara). Real-time PCR was performed using SYBR Premix ExTaq kit (Takara) on ABI PRISM 7900 Sequence Detection System (Applied Biosystems), all according to the manufacturer’s instructions. Detected gene expression was normalized to the housekeeping gene (Rpl13a). The real-time PCR primers were shown in Supplementary Table 1.

### Cell death assays

Cell death was determined by detecting the level of released cytoplasmic enzyme lactate dehydrogenase (LDH) or propidium iodide (PI)-positive cells through FACS. For the LDH release assay, cells were seeded in 96-well plates. Before stimulation, media was changed with OPTI MEM. Cells were treated as indicated and the supernatant was collected to detect the released LDH using the CytoTox96 LDH release kit (Promega), according to the manufacturer’s protocol. Total LDH was produced by adding lysis buffer to liberate all cytoplasmic LDH. Cell viability was determined with the percentage of total LDH. For PI staining, peritoneal macrophages were seeded in 12-well plates and then treated as indicated. The cells were collected with EDTA-trypsin and washed in PBS. Peritoneal macrophages were stained with 2.5μg/ml PI in sterile PBS for 20min. After that, the cells were centrifuged and washed in sterile PBS. PI-positive cells were analyzed using a FACScan flow cytometer (BD Biosciences).

### Immunoprecipitation and immunoblot analysis

Immunoprecipitation and immunoblot analysis were performed as described previously^35^. Briefly, the cells were treated as indicated and washed with cold PBS. The cells were harvested with lysis buffer (50 mM Tris-HCl (pH 7.5), 150 mM NaCl, 1.0% Triton X-100, 1 mM EDTA, and 10% glycerol containing protease inhibitors). Cell extracts were left on ice for 30 min and centrifuged at 13,000 r.p.m for 30 min. Cell lysates were incubated with 2μg of anti-RIPK1 antibody or 2μg of normal mouse IgG antibody (Santa Cruz) overnight at 4°C and then incubated with 15μl of protein A beads for an additional 3h at 4°C. The beads were washed with lysis buffer three times and then boiled in 20μl of 1×SDS loading buffer for 10 min. Samples were loaded on SDS-PAGE gels followed by electroblotting onto PVDF membranes (Millipore). To determine protein levels, immunoblot was performed using the following antibodies: anti-RIPK3 (2283) from Pro Sci; anti-pMLKL (ab196436) from Abcam; anti-RIPK1 (610459) from BD Biosciences; anti-pP38 (9211S), anti-JNK (9252), anti-pERK1/2 (4370), anti-pRIPK1 (S166) (31122S), anti-pJNK (9251) from Cell Signaling, anti-Tim23 (11123-1-AP) from Proteintech, anti-GAPDH (sc-32233) from Santa Cruz, and anti-TRIF (657102) from Biolegend.

### RIPK3 and MLKL oligomerization detection

Peritoneal macrophages were seeded in 12-well plates and treated as indicated. Cells were washed with cold PBS and harvested with 2×DTT-free sample buffer (125mM Tris-Cl pH 6.8, SDS 4%, glycerol 20%, and bromophenol blue 0.02%). Protein samples were loaded on SDS-PAGE gels followed by electroblotting onto PVDF membranes (Millipore). Immunoblot analysis was performed with RIPK3 and p-MLKL antibody.

### Histology

Mice treated with TNF or infected with *Staphylococcus aureus* were sacrificed. Liver, ileum, and cecum from mice treated with TNF and lung from mice infected with *Staphylococcus aureus* were fixed in 4% paraformaldehyde for at least 2 days. Fixed samples were embedded into paraffin and sliced into 5-μm sections. Five-micrometer sections were stained with H&E, according to the standard procedures described previously^34^. The images were captured with a Leica FDM2500 microscope.

### TNF-induced SIRS model

Eight to ten weeks old C57BL/6 female mice were used for experiments. Mouse recombinant TNF, DMSO, and SP600125 were diluted in endotoxin-free PBS. Mice were injected i.p. with DMSO or SP600125 for 30 min. And then mice were injected intravenously (i.v.) with 15μg of TNF. Mortality of mice was monitored after TNF injection. Plasma samples and tissue samples of ileum, liver, and cecum were collected at indicated times after injection.

### *Staphylococcus aureus* infection

*Staphylococcus aureus* USA300 was from ATCC. Eight to ten weeks old C57BL/6 female mice were injected intraperitoneally with DMSO or SP600125 for 1h. And then mice were intranasally infected with 10^7^ colony-forming units (CFU)/mouse *Staphylococcus aureus*. Twenty-four hours later, lungs from infected mice were removed for histology and measured with CFU. Homogenized lungs were diluted with sterile PBS and plated on Luria Bertani (LB) agar plates. CFU were counted after 12h on LB agar plates at 37°C.

### Statistical analysis

Prism software (GraphPad Software) was used to perform statistical analysis and graph development. Two-tailed Student’s *t* test was used to compare differences between two groups. Survival curves were presented using Kaplan–Meier method and significance was calculated by log-rank (Mantel–Cox) test. Statistical significance was defined as *P* < 0.05. *P <  0.05, **P <0.01, and ***P < 0.001.

## Results

### RIPK1 and RIPK3 kinases promote JNK and P38 activation and downstream pro-inflammatory cytokines expression in the absence of caspase 8 activation

Previous studies have shown that RIPK1 and RIPK3 kinases promoted cell death-independent pro-inflammatory role induced by LPS, and the MAPKs ERK1/2 contribute to RIPK1- and RIPK3-induced pro-inflammatory cytokines production^36,37^. Therefore, we wonder whether other MAPKs (P38 and JNK) also participate in the RIPK1- and RIPK3-dependent pro-inflammatory signaling pathways. We also found that, compared to the treatment of LPS or poly I:C alone, LPS or poly I:C in the presence of caspases pan-inhibitor zVAD significantly increased the pro-inflammatory cytokines (*IL-6, KC, and IFNβ*) production in peritoneal macrophages (Supplementary Fig. 1a). The amplified inflammatory cytokines were reduced in the presence of P38 inhibitor or JNK inhibitor (Supplementary Fig. 1b,c), indicating that P38 and JNK might also be essential for RIPK1- and RIPK3-mediated inflammatory signaling pathway.

**Fig. 1:**
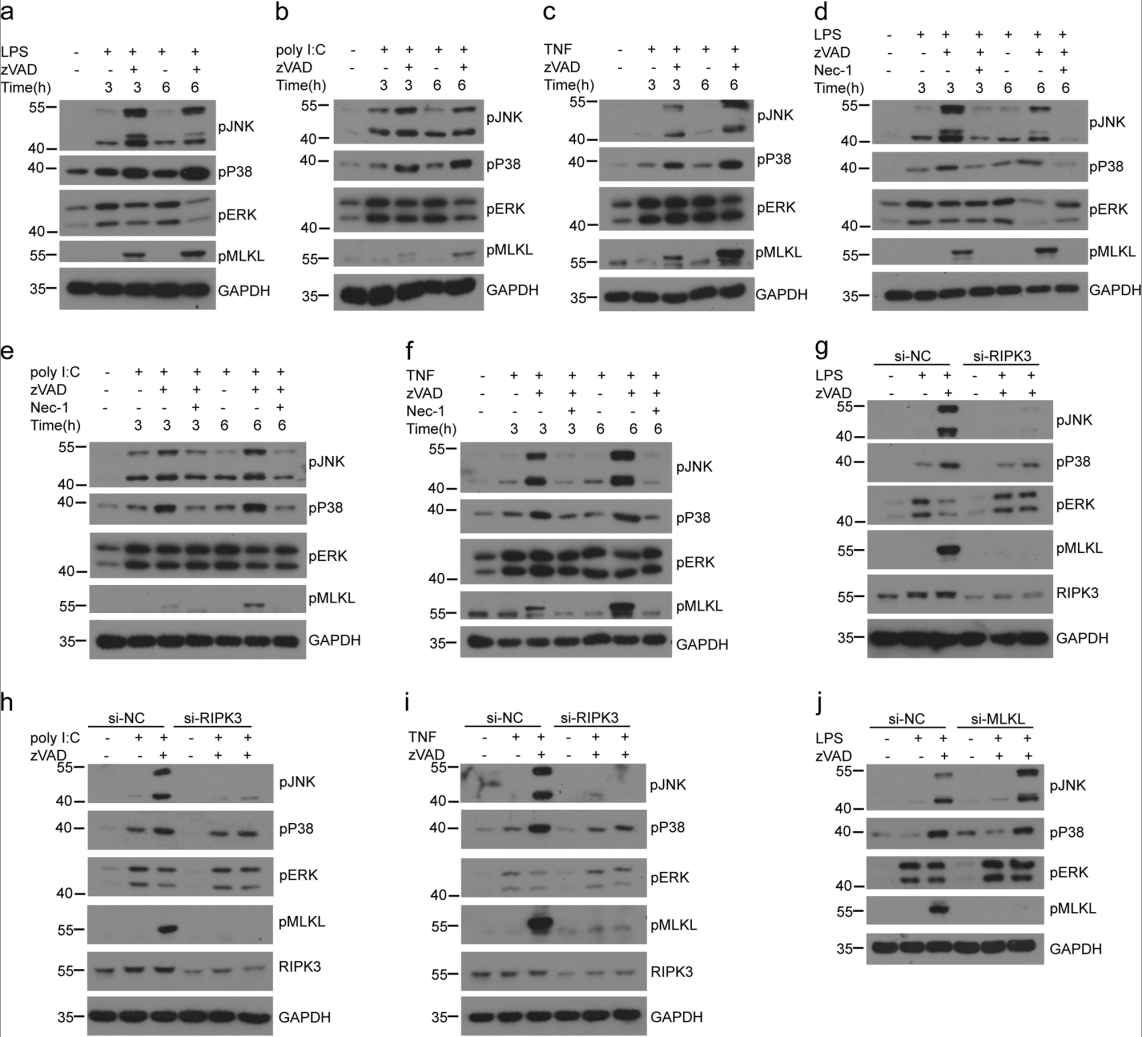
Necroptosis-activated RIPK1 and RIPK3 promote cell-death-independent P38 and JNK activation in macrophages. **a**–**c** Peritoneal macrophages from wild-type mice were left untreated or pretreated with zVAD (20μM) for 30min and then treated with LPS (25ng/ml) (**a**), poly I:C (30μg/ml) (**b**), or recombinant mouse TNF (50ng/ml) (**c**) for the indicated times. MAPKs activation and MLKL phosphorylation were assessed by immunoblotting with the indicated antibodies. **d**–**f** Peritoneal macrophages were left untreated or pretreated with zVAD or zVAD+Nec-1 (30μM) for 30min and then treated with LPS (**d**), poly I:C (**e**), or TNF (**f**) for the indicated times. MAPKs activation and MLKL phosphorylation were assessed by immunoblotting with the indicated antibodies. **g**–**i** Peritoneal macrophages were transfected with scramble siRNA (si-NC) or RIPK3 siRNA (si-RIPK3) for 3 days. Then the cells were left untreated or pretreated with zVAD for 30 min followed by LPS (**g**), poly I:C (**h**), or TNF (**i**) treatment for an additional 3h. Lysates were analyzed by immunoblotting with the indicated antibodies. **j** Peritoneal macrophages were transfected with si-NC or si-MLKL for 3 days, and then untreated or treated with zVAD for 30 min followed by LPS treatment for 3h. Lysates were analyzed by immunoblotting with the indicated antibodies. Data are representative of at least three independent experiments

To further confirm the roles of P38 and JNK in RIPK1- and RIPK3-induced inflammatory signaling pathway, we analyzed the activation of P38 and JNK and found that LPS plus zVAD induced more P38 and JNK activation compared with those induced by LPS alone (Fig. 1a). We also checked the necroptosis settings induced by poly I:C or TNF and found the similar results as in LPS-induced necroptosis (Fig. 1b, c). Previous studies showed that the amplified inflammation was dependent on RIPK1 and RIPK3^36^. To identify whether increased P38 and JNK activation is dependent on RIPK1 and RIPK3, we used the RIPK1 kinase inhibitor necrostatin-1 (Nec-1) and a specific short- interfering RNA (siRNA) targeting RIPK3. We found that Nec-1 treatment abolished P38 and JNK activation induced by LPS, poly I:C, or TNF plus zVAD in peritoneal macrophages (Fig. 1d–f). Similarly, RIPK3 siRNA also impaired the phosphorylation of JNK and P38 in peritoneal macrophages stimulated with LPS, poly I:C, or TNF plus zVAD (Fig. 1g–i). As previous work has shown that RIPK1- and RIPK3-induced inflammation is independent on cell death from the results of MLKL-deficient macrophages^36^, we also found that knockdown MLKL could not change the activation of JNK and P38 in peritoneal macrophages treated with LPS plus zVAD (Fig. 1j). Overall, these data indicate that RIPK1 and RIPK3 kinases can promote cell-death-independent JNK and P38 activation in the absence of caspase activities.

### Inhibition of JNK activation prevents TNF- and TLRs-triggered necroptosis

**Fig. 2:**
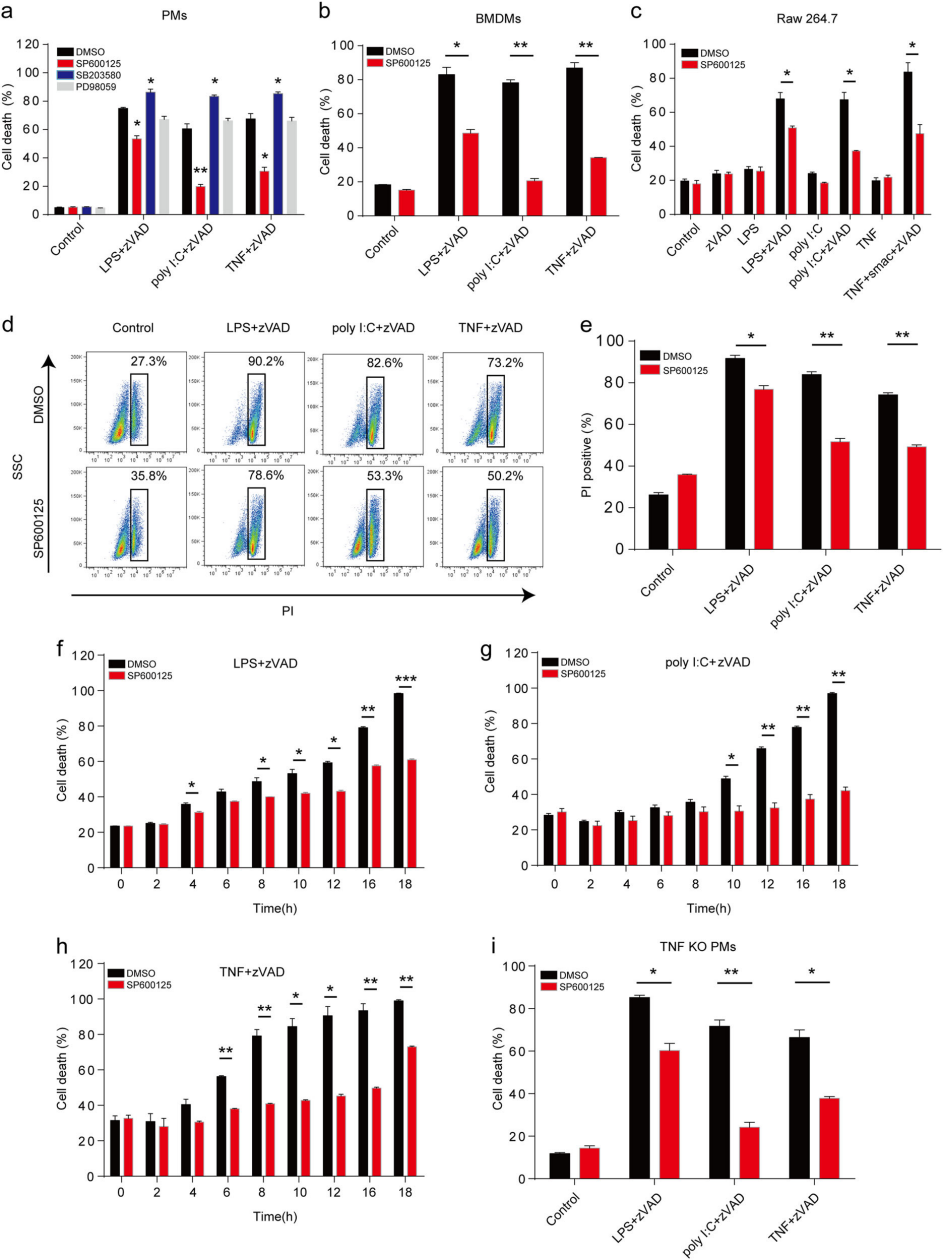
Inhibition of JNK with SP600125 restricts both TNF-induced and TLRs-induced necroptosis in macrophages. **a** Peritoneal macrophages were pretreated with zVAD, DMSO, SP600125 (20μM), SB203580 (10μM), or PD98059 (10μM) for 30 min, followed by LPS, poly I:C, or TNF treatment for an additional 12h. Cell death was determined by released lactate dehydrogenase (LDH). **b**, **c** Bone marrow-derived macrophages (BMDMs) (**b**) and Raw 264.7 cells (**c**) were pretreated with zVAD, DMSO, smac, or SP600125 for 30 min, followed by LPS, poly I:C, or TNF treatment for an additional 12h. Cell death was determined by released LDH. **d**, **e** Peritoneal macrophages were stimulated as in **b** for 12h and then were stained with propidium iodide (PI). The cells were analyzed with fluorescence-activated cell sorting (FACS). Representative plots of data were shown in **d**. The percentage of PI-positive cells was presented in **e**. **f**–**h** Peritoneal macrophages were treated as in **b** for the indicated time points. The cell death of peritoneal macrophages triggered by LPS (**f**), poly I:C (**g**), or TNF (**h**) was measured by released LDH. **i** Peritoneal macrophages from wild-type and TNF-deficient mice were treated as in **b** for 12h. Cell death was determined by released LDH. Data are representative of at least three independent experiments and shown as mean±SEM in graphs **a**–**c** and **e–i**. * *p*< 0.05, **p < 0.01, and *** *p*< 0.001 by Student’s *t* test

Necroptosis is tightly regulated to avoid adverse cell death and inflammation, P38 activates downstream MK2 kinase to phosphorylate RIPK1 and consequently inhibit RIPK1-dependent apoptosis and TNF-induced necroptosis^23–25^. Therefore, we wanted to check whether JNK could regulate necroptosis signaling pathway as P38. We used specific MAPK inhibitors (SP600125, SB203580, and PD98059) to pretreat peritoneal macrophages and then induced necroptosis with TNF or TLRs. As expected, P38 inhibition sensitized macrophages to necroptosis induced by TNF or TLRs (Fig. 2a). However, JNK inhibition dramatically restrained TNF- or TLRs-induced necroptosis and ERK inhibition showed no effect on the necroptosis (Fig. 2a). To confirm the role of JNK in necroptosis, we performed the cell survival experiments in both bone marrow-derived macrophages (BMDMs) and Raw 264.7 cells and found that JNK inhibition indeed reduced the TNF- or TLRs-triggered necroptosis in both cells (Fig. 2b, c). Similarly, peritoneal macrophages treated with JNK inhibitor also had more survival cells with the intact cell morphology (Supplementary Fig. S2). To confirm the findings further, FACS was used to evaluate the role of JNK in necroptosis. We found that, compared with indicated necroptotic control settings, JNK inhibitor treatment significantly decreased the percentage of PI-positive macrophages (Fig. 2d, e). In addition, our data showed that JNK can inhibit the macrophage necroptosis in an early period of time (Fig. 2f–h). Considering that JNK are required for the production of inflammatory cytokines including TNF, it is possible that the effect of JNK inhibition on necroptosis is due to the reduced autocrine production of TNF from macrophages. To exclude this possibility, we treated the TNF-deficient macrophages with SP600125 under necroptotic conditions. However, JNK inhibition still reduced the TNF- or TLRs-induced necroptosis in TNF-deficient macrophages as in wild-type macrophages (Fig. 2i). Collectively, these data imply that JNK kinases activity is directly required for the necroptosis induced by TNF and TLRs.

### Necrosome formation and MLKL activation are compromised in the presence of JNK inhibitor

**Fig. 3:**
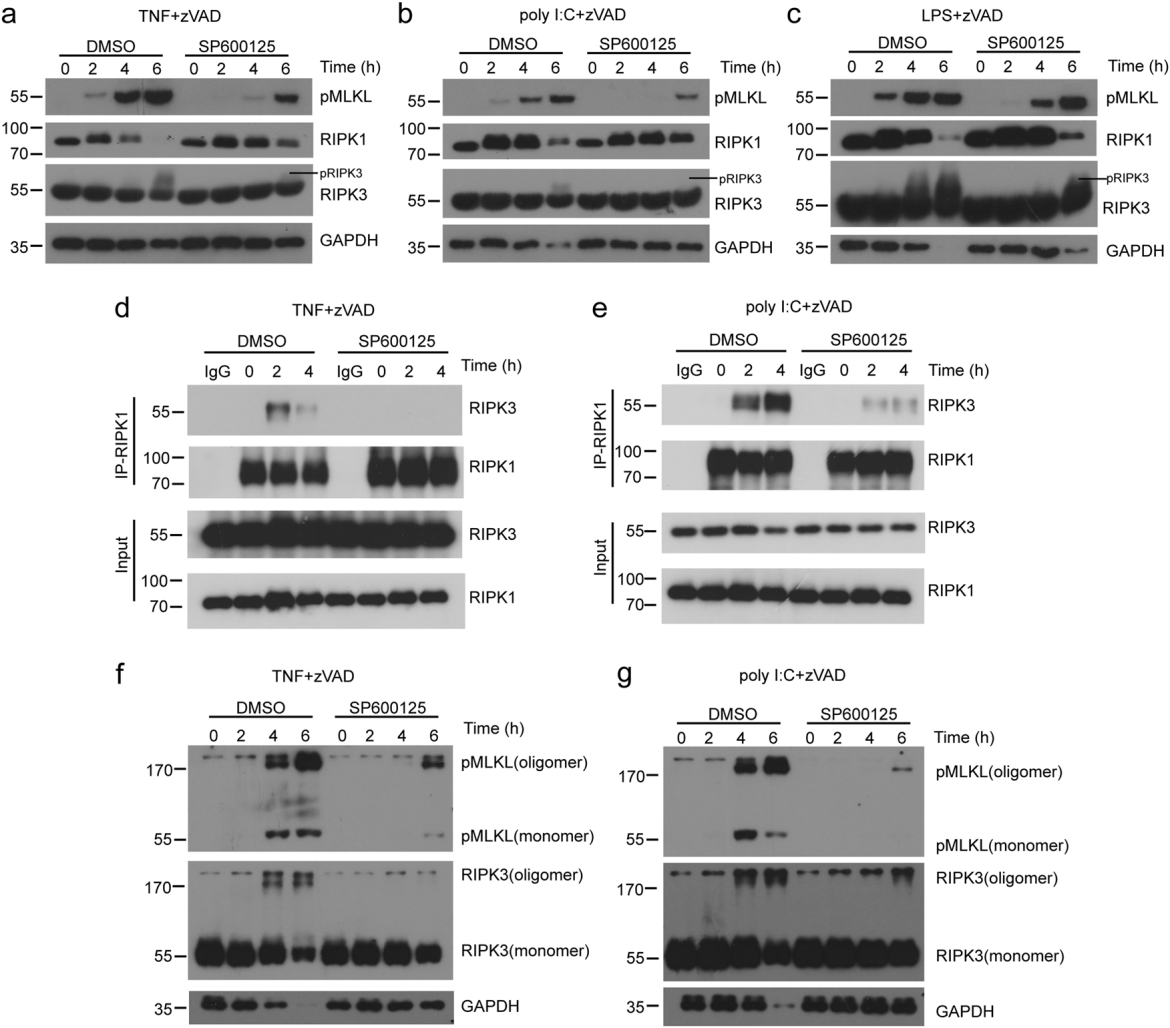
Inhibition of JNK using SP600125 reduces necrosome formation in macrophages. (**a–c**) Peritoneal macrophages were pretreated with zVAD, DMSO, or SP600125 for 30 min, followed by TNF (**a**), poly I:C (**b**), or LPS (**c**) treatment for the indicated times. Lysates were analyzed by immunoblotting with the indicated antibodies. **d**, **e** Immunoblot analysis with indicated antibodies of RIPK1 or mouse IgG immunoprecipitates and total lysates from peritoneal macrophages treated with TNF+zVAD (**d**) and poly I:C+zVAD (**e**) for indicated periods of time. **f**, **g** Peritoneal macrophages were treated by TNF (**f**) or poly I:C (**g**) as in **d** or **e**. Lysates were analyzed by immunoblotting with antibodies against pMLKL, RIPK3, or GAPDH. Data are representative of at least three independent experiments

To determine how JNK regulates the necroptotic signaling pathway, we further examined the necroptotic complex formation. Inhibition of JNK activation reduced the levels of phosphorylation of MLKL (pMLKL), as well as pRIPK3 in peritoneal macrophages stimulated by TNF and zVAD (Fig. 3a). Similar results were observed in peritoneal macrophages treated with LPS plus zVAD or poly I:C plus zVAD (Fig. 3b, c). In Raw 264.7 cells, we also found that treatment of JNK inhibitor dramatically reduced pMLKL levels (Supplementary Fig. S3). We immunoprecipitated endogenous RIPK1 with anti-RIPK1 antibody and found that the level of RIPK3 was increased in peritoneal macrophages by TNF- or poly I:C-induced necroptosis (Fig. 3d, e). However, peritoneal macrophages treated with JNK inhibitor had a dis-association of RIPK1 with RIPK3 (Fig. 3d, e). We found that oligomerization of RIPK3 and pMLKL was induced in control peritoneal macrophages treated with TNF or poly I:C plus zVAD, while the oligomerization of RIPK3 and pMLKL was significantly suppressed by JNK inhibition (Fig. 3f, g). Together, these results suggest that JNK kinase activities are required for necrosome formation and oligomerization of RIPK3 and MLKL.

### Loss of JNK suppresses TNF-induced necroptosis but promotes TLRs-triggered necroptosis

**Fig. 4:**
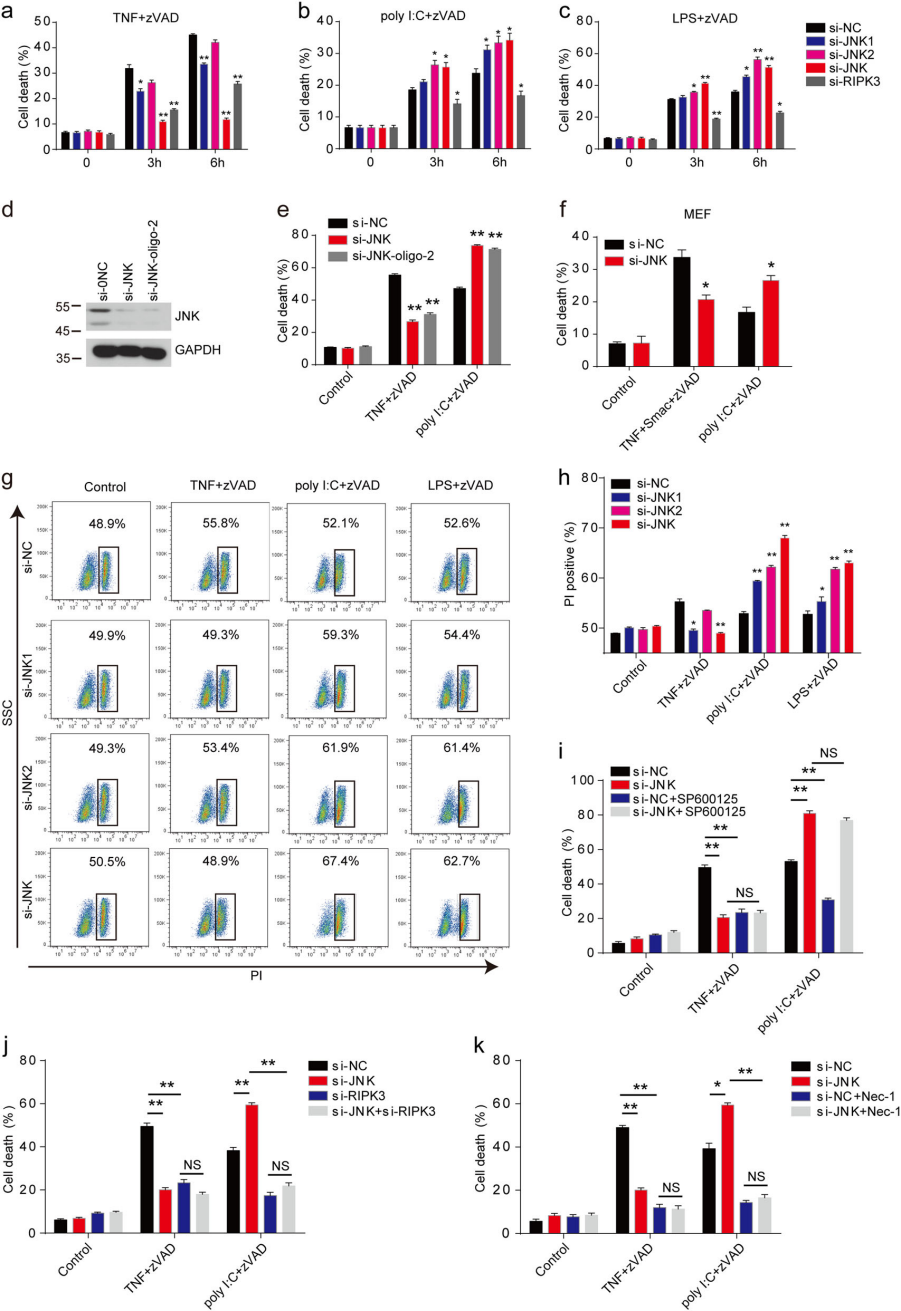
JNK knockdown attenuates TNF-induced necroptosis but augments TLRs-induced necroptosis. **a**–**c** Peritoneal macrophages were transfected with indicated siRNA for 3 days. Cells were then treated with TNF+zVAD (**a**), poly I:C+zVAD (**b**), or LPS+zVAD (**c**) for the indicated time. Cell death was determined by released LDH. **d** Peritoneal macrophages were transfected with indicated siRNA oligos for 3 days. Lysate were analyzed by immunoblotting with antibodies against JNK or GAPDH. **e** Cells as in **d** were treated with TNF+zVAD, poly I:C+zVAD for 6 h, and cell death was determined by released LDH. **f** MEF cells were transfected with control siRNA or JNK-specific siRNA for 3 days, and then treated with TNF+Smac+zVAD or poly I:C+zVAD for 12 h. Cell death was measured by released LDH. **g**, **h** Peritoneal macrophages were transfected with indicated siRNA for 3 days. Cells were then treated with TNF+zVAD, LPS+zVAD, or poly I:C+zVAD for 4 h. The cells were stained with PI, and followed by FACS analysis. Representative plots of data are shown in **g**. Percentage of PI-positive cells is presented in **h**. **i** Peritoneal macrophages were transfected with si-NC or si-JNK for 3 days and then pretreated with DMSO, SP600125, or zVAD for 30 min, followed by poly I:C or TNF treatment for 6 h. Cell death was measured by released LDH. **j** Peritoneal macrophages were transfected with indicated siRNA for 3 days and then pretreated with zVAD for 30 min, followed by poly I:C and TNF treatment for 6 h. Cell death was measured by released LDH. **k** Peritoneal macrophages were transfected with si-NC or si-JNK for 3 days and then pretreated with Nec-1 or zVAD for 30 min, followed by poly I:C and TNF treatment for 6 hours. Cell death was measured by released LDH. Data are representative of at least three independent experiments and shown as mean±SEM in graphs **a–c**, **e**, **f**, and **h–k**. *p < 0.05, **p < 0.01 by Student’s *t* test. JNK: JNK1+JNK2, NS: no significance

To confirm the results from kinase inhibitors, we used the JNK-specific short-interfering RNA (siRNA) to interfere the expression of the ubiquitously expressed JNK1 and JNK2. Loss of JNK1 significantly suppressed the cell death of peritoneal macrophages in TNF-induced necroptosis, while JNK2 absence had only a weak suppressive effect in TNF-induced necroptosis (Fig. 4a). However, we found that loss of both JNK1 and JNK2 had a much more suppressive effect than the single suppression of JNK1 or JNK2 expression (Fig. 4a), indicating that JNK1 and JNK2 played redundant roles in TNF-induced necroptosis. We next examined the LPS- or poly I:C-induced necroptosis in the JNK1 or JNK2 knockdown macrophages. Unexpectedly, loss of JNK1 and JNK2 sensitized macrophages to LPS- or poly I:C-induced necroptosis, which opposes the results of JNK inhibitor in TLRs-induced necroptosis (Fig. 4b, c). To exclude the off-target effects of si-JNK oligo, we used another si-JNK oligo (si-JNK-oligo-2) and found the consistent results in peritoneal macrophages (Fig. 4d, e). We also found that the necrotic cell death of JNK-depleted MEF cells induced by TLRs was accelerated and necrotic cell death induced by TNF was inhibited (Fig. 4f). Moreover, we found similar opposite results by analyzing the PI-positive necroptotic macrophages (Fig. 4g, h). A few reports showed that JNK inhibitor SP600125 may have an off-target effect to interfere with cellular processes^38,39^. To exclude the off-target effect of the JNK inhibitor, we treated the knockdown macrophages with JNK inhibitor SP600125, and found that SP600125 showed no effect on both TNF- and TLR-induced necroptosis in these cells (Fig. 4i), which indicated that the potential off-target effect did not contribute to the necroptosis phenotype we observed above. The similar contradictory phenotypes were also observed from the RIPK1 kinase, which played positive roles in TNF-induced necroptosis but negatively regulated poly I:C-induced necroptosis through its scaffolding activity^40,41^. Thus, we investigated whether JNK-loss-augmented necroptosis was dependent on RIPK1 and RIPK3. Indeed, inhibition of RIPK1 activity by Nec-1 and siRNA targeting RIPK3 blocked the JNK-loss-augmented necroptosis induced by poly I:C (Fig. 4j, k). Therefore, our data suggested that JNK regulated necroptosis through both their kinase and scaffolding activities.

### Loss of JNK inhibits TNF-induced necrosome formation but promotes TLRs-triggered necrosome formation

**Fig. 5:**
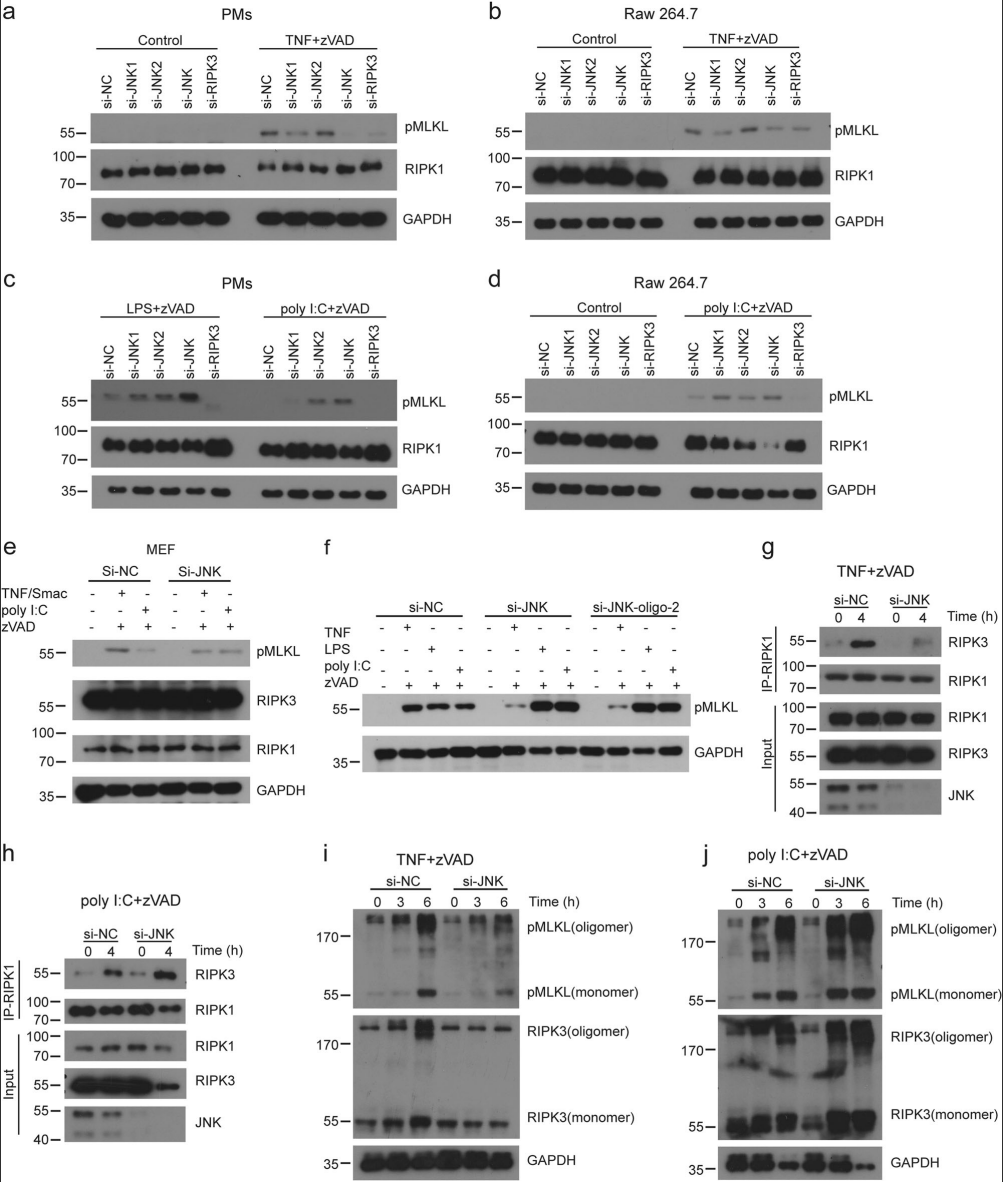
JNK knockdown attenuates TNF-induced necrosome formation but augments TLRs-induced necrosome formation. **a**–**d** Peritoneal macrophages (**a**, **c**) or Raw 264.7 cells (**b**, **d**) were transfected with indicated siRNA for 3 days and then treated with TNF+zVAD, LPS+zVAD, or poly I:C+zVAD for 3 h. MLKL phosphorylation was assessed by immunoblotting with the indicated antibodies. (**e**) MEF cells were transfected with indicated siRNA for 3 days and then treated with TNF+Smac+zVAD, or poly I:C+zVAD for 3 h. MLKL phosphorylation was assessed by immunoblotting with the indicated antibodies. **f** Peritoneal macrophages were transfected with control siRNA, si-JNK, or si-JNK-oligo-2 for 3 days, and then treated with TNF+zVAD, LPS+zVAD, or poly I:C+zVAD for 3 h. MLKL phosphorylation was assessed by immunoblotting with the indicated antibodies. **g**, **h** Peritoneal macrophages were transfected with si-NC or si-JNK for 3 days and then treated with TNF+zVAD (**g**) or poly I:C+zVAD (**h**) for 4 h. The cell lysates were immunoprecipitated using anti-RIPK1 antibody and then analyzed by immunoblotting with indicated antibodies. **i**, **j** Peritoneal macrophages were transfected with each siRNA for 3 days and then treated with TNF+zVAD (**i**) or poly I:C+zVAD (**j**) for the indicated times. Lysates were analyzed by immunoblotting with indicated antibodies. Data are representative of at least three independent experiments

Next, we investigated the necroptotic signaling pathway in the absence of JNK protein by siRNA interference. Our results showed that JNK proteins, especially JNK1 proteins, were required for TNF-induced phosphorylation of MLKL in peritoneal macrophages (Fig. 5a) and Raw 264.7 cells (Fig. 5b). However, in poly I:C-induced necroptosis, absence of JNK led to overphosphorylation of MLKL in peritoneal macrophages (Fig. 5c) and Raw 264.7 cells (Fig. 5d). Besides macrophages, we also obtained similar results in MEF cells (Fig. 5e). To exclude off-target effects of si-JNK oligo, we used another si-JNK oligo (si-JNK-oligo-2) and found the consistent results with si-JNK (Fig. 5f). We analyzed the formation of RIPK3–RIPK1 necrosome complexes and found that loss of JNK decreased the formation of TNF-induced necrosome complexes while poly I:C-induced necrosome formation was increased (Fig. 5g, h). Consistently, loss of JNK attenuated oligomer formation of RIPK3 and pMLKL induced by TNF, while it promoted oligomer formation triggered by poly I:C (Fig. 5i, j). Collectively, these data indicated that JNK distinguishingly regulated necrosome formation induced by TNF or poly I:C.

### JNK, which is recruited by RIPK1 to necrosome complexes, promotes the autophosphorylation of RIPK1 and inhibits the oligomerization of TRIF during necroptosis

JNK has been reported to promote the production of cytotoxic reactive oxygen species (ROS), which contributes to TNF-induced necrosis^42^. Then we investigated whether JNK regulated TNF- and TLRs-induced necroptosis through ROS production. We used protonophore carbonylcyanide m-chlorophenylhydrazone (CCCP) to deplete the mitochondria of peritoneal macrophages^43^. The CCCP-treated macrophages had almost no mitochondria compared with untreated cells (Supplementary Fig. 4a). Meanwhile, we found that CCCP-treated macrophages have compromised, especially TNF-induced necroptosis (Supplementary Fig. 4b). However, our results showed that inhibition of JNK with SP600125 still restrained the necroptosis in CCCP-treated macrophages (Supplementary Fig. 4c-e), as well as reduced pMLKL level in these macrophages (Supplementary Fig. 4f). On the other hand, we used the ROS scavenger butylated hydroxyanisole (BHA) and found that inhibition of JNK with SP600125 still decreased the necrototic cell death in BHA-treated macrophages (Supplementary Fig. 4g). Collectively, our data suggested that JNK regulated necroptosis independent of mitochondrial ROS.

**Fig. 6:**
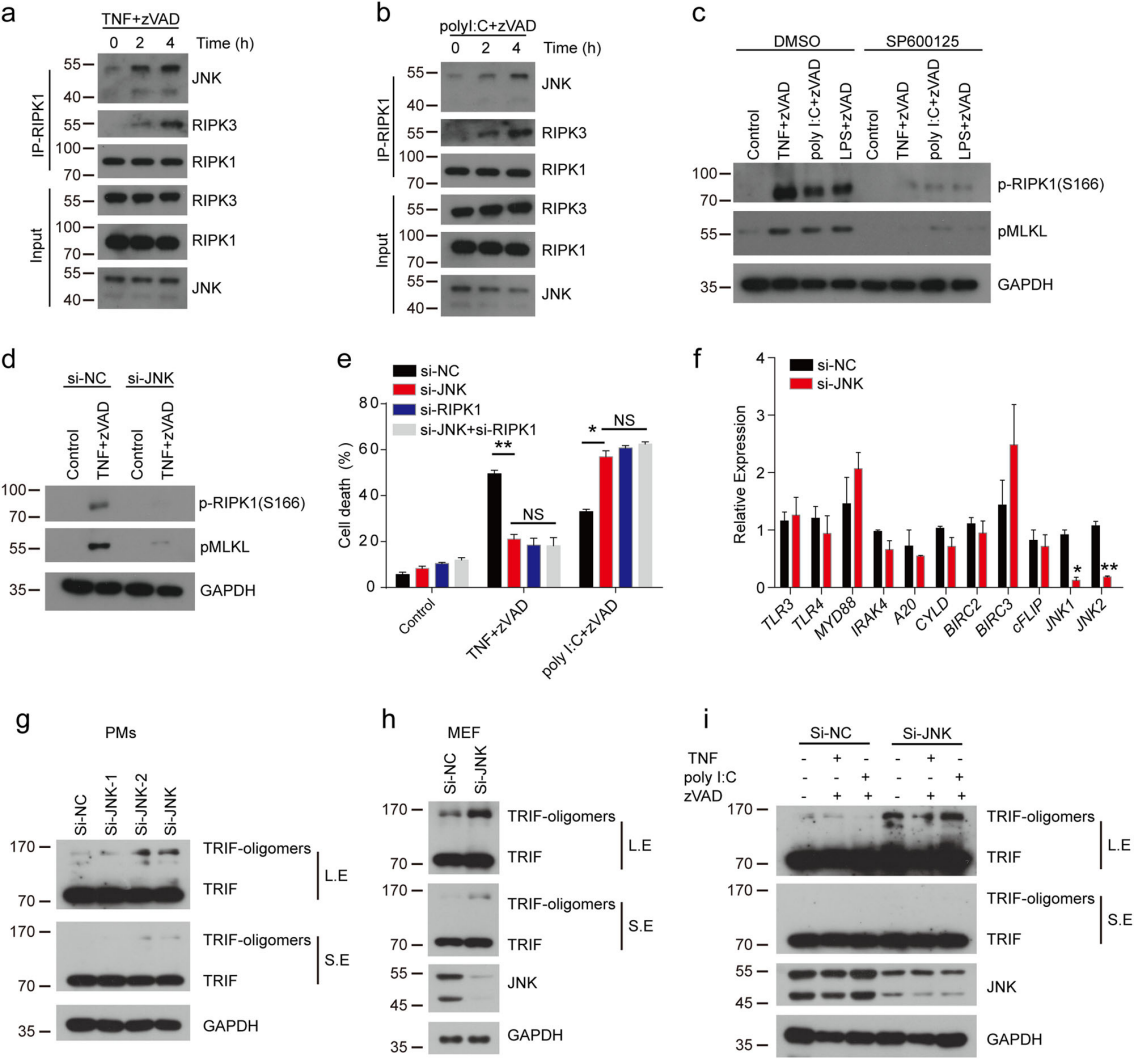
JNK is recruited to necrosome and then regulates necroptosis through RIPK1 and TRIF. **a**, **b** Peritoneal macrophages were treated with TNF+zVAD (**a**) or poly I:C+zVAD (**b**) for the indicated time. Cell lysates were immunoprecipitated with anti-RIPK1 antibody and then analyzed by immunoblotting using the indicated antibodies. **c** Peritoneal macrophages were pretreated with zVAD, DMSO, or SP600125 for 30 min, followed by TNF, poly I:C, or LPS treatment for 3 h. Lysates were analyzed by immunoblotting with indicated antibodies. **d** Immunoblot analysis of peritoneal macrophages transfected with indicated siRNA for 3 days and then treated with TNF+zVAD for 3h. **e** Peritoneal macrophages were transfected with indicated siRNA for 3 days and then treated with TNF+zVAD and poly I:C+zVAD for 6h. Cell death was measured by released LDH. **f** Peritoneal macrophages transfected with control siRNA and JNK-specific siRNA for 3 days and then the expression of TLR3, TLR4, Myd88, IRAK4, A20, cyld, BIRC2, BIRC3, cFlip, JNK1, and JNK2 determined via real-time PCR. **g**, **h** Immunoblot analysis of TRIF in peritoneal macrophages (**g**) or MEF cells (**h**) transfected with indicated siRNA for 3 days. **i** Immunoblot analysis of TRIF in peritoneal macrophages transfected with indicated siRNA for 3 days, and then treated with TNF+zVAD or poly I:C+zVAD for 3 h. Data are representative of at least three independent experiments and shown as mean±SEM in graph **e**, **f**. *p < 0.05 by Student’s *t* test. NS no significance, LE long exposure, SE short exposure

Our results indicated that JNK regulated necroptosis by their kinase activities and scaffolding activities. Thus, we investigated whether JNK directly regulated necroptosis. We immunoprecipitated RIPK1 and found that the association of JNK with RIPK1 was increased in the process of necroptosis triggered by TNF or poly I:C (Fig. 6a, b), indicating that JNKs were recruited to necrosome by RIPK1 to regulate necrosome formation. Autophosphorylation of RIPK1 has been shown that is responsible for necroptosis. We found that inhibition of JNK with SP600125 dramatically blocked the autophosphorylation of RIPK1 (Fig. 6c). Similarly, loss of JNK by siRNA also suppressed autophosphorylation of RIPK1 in TNF-induced necroptosis (Fig. 6d). Previous work reported that RIPK1 showed double-edge functions to regulate necroptosis^40,41^.Therefore, we suspected that JNKs were likely to function similarly in a RIPK1-dependent way to regulate necroptosis. We found that, as loss of JNK, RIPK1 depletion by siRNA showed a similar distinguishable phenotype in TNF- and poly I:C- induced necroptosis (Fig. 6e). However, loss of JNK showed no effect on the necroptosis in RIPK1-silenced cells (Fig. 6e), which implied that JNK regulated necroptosis through RIPK1. To further investigate the mechanism of JNK that negatively regulate TLRs-induced necroptosis, we checked the expression of genes (TLR3, TLR4, Myd88, IRAK4, A20, Cyld, BIRC2, BIRC3, and cFLIP), which were important molecules of TLRs signaling pathway, in JNK-depleted macrophages and found that they exhibited no significant difference compared with control macrophages (Fig. 6f). In TLRs-induced necroptosis, TRIF recruits RIPK1 and RIPK3 through RHIM domain and is indispensible for TLRs-induced necroptosis^44^, so we asked whether JNK negatively regulated TLRs-induced necroptosis by regualting the expression of TRIF. We compared the abundance of TRIF in JNK1- and JNK2-depleted macrophages. JNK depletion in macrophages resulted in the accumulation of high-molecular-weight oligomers of TRIF (Fig. 6g). We also observed similar results in JNK-depleted MEF cells (Fig. 6h). In the necroptotic process, JNK knockdown also increased the abundance of TRIF oligomers (Fig. 6i). Notably, oligomerization of TRIF via RHIM domain is known to be critical for downstream signaling transduction^45^. Consistently, we observed more type I interferons in JNK-deficient macrophages (Supplementary Fig. 4h). Therefore, our results showed that JNK inhibited TLRs-induced necroptosis by suppressing the oligomerization of TRIF.

### Mice pretreated with JNK inhibitor show protection from TNF-induced SIRS and *Staphylococcus aureus*-mediated lung damage

**Fig. 7:**
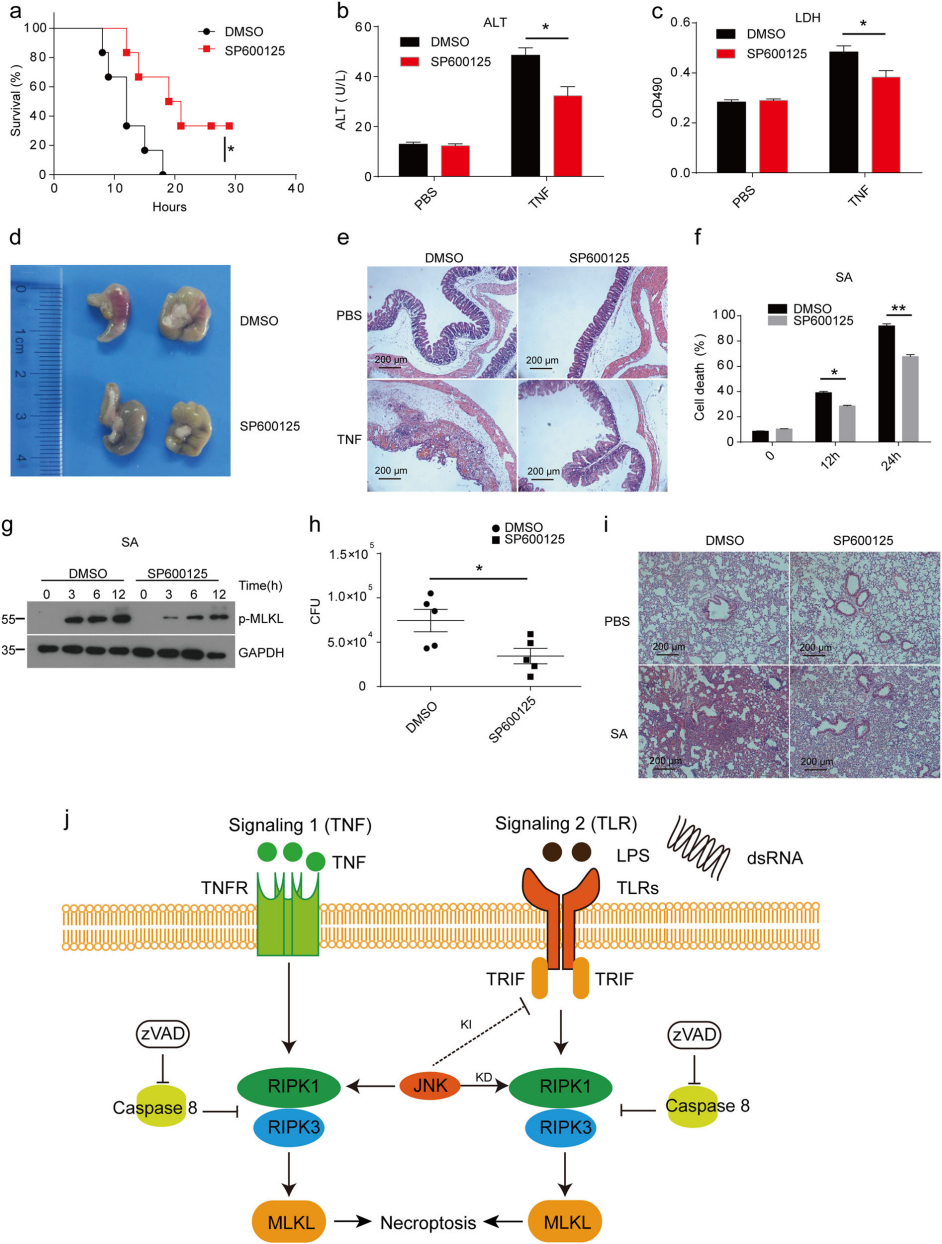
JNK inhibition with SP600125 protects mice from TNF-induced SIRS and *Staphylococcus aureus*-mediated lung damage. **a** Wild-type mice were injected i.p. with DMSO (*n* = 6) or SP600125 (*n* = 6) for 30 min prior to a challenge with 15μg of TNF per mice through the tail vein. Mouse survival was monitored. **b**, **c** Plasma samples of mice treated with DMSO or SP600125 were collected 12h after challenge with TNF or PBS and analyzed for activities of ALT (**b**) and LDH (**c**). **d** Macroscopic view of the representative cecums from mice treated as in **a** for 12h. **e** H&E histology of the representative cecums from mice treated as in **b** and **c**. Photomicrographs of histology are shown at 100× magnification. **f** Peritoneal macrophages were pretreated with DMSO or SP600125 for 30 min and then treated with MOI 1 *Staphylococcus aureus* (SA) for the indicated time. Cell death was measured by released LDH. **g** Immunoblot analysis of peritoneal macrophages pretreated with DMSO or SP600125 for 30 min and then treated with MOI 1 SA for the indicated time. **h** Wild-type mice were treated with DMSO or SP600125 and then infected with SA. CFU in the lung was assayed 24h after infection. **i** H&E histology of the representative lungs from mice treated with DMSO+PBS, SP600125+PBS, DMSO+SA, and SP600125+SA for 24h. Photomicrographs of histology are shown at ×100 magnification. **j** Model of the kinase-dependent (KD) and -independent (KI) functions of JNK in necroptosis. Data are representative of at least three independent experiments and shown as mean±SEM in graphs **b**, **c**, **f**, and **h**. *p < 0.05, **p < 0.01 by Student’s *t* test

Necroptotic process is essential for TNF-induced systemic inflammatory response syndrome (SIRS)^14^. To determine the function of JNK in necroptosis in vivo, we injected JNK inhibitor into wild-type mice following TNF treatment. Mice treated with the inhibitor were more resistant to TNF-induced SIRS than the control group (Fig. 7a). Consistently, the releasing of plasma (alanine aminotransferase) ALT and LDH from damaged tissue was significantly lower in mice treated with JNK inhibitor than in control mice (Fig. 7b, c). In addition, we can observe alleviated cecum damage in the JNK inhibition mice group (Fig. 7d, e), although, we did not detect an obvious difference in liver and ileum between groups (Supplementary Fig. 5a,b). Recently, it has been reported that *Staphylococcus aureus* can trigger necroptosis through bacterial toxins, which contributed to *Staphylococcus aureus*-mediated lung damage^6^. Therefore, we investigated the role of JNK in *Staphylococcus aureus*-mediated necroptosis and lung damage in vivo. We found that JNK inhibition limited *Staphylococcus aureus*-induced cell death as well as activation of MLKL (Fig. 7f, g). However, SP600125 did not affect the growth of *Staphylococcus aureus* in vitro (Supplementary Fig. 5c). Mice treated with JNK inhibitor presented lower lung damage and bacterial burden in their lung tissues compared to the control mice (Fig. 7h, i). Overall, our data demonstrated that JNK kinase activity was essential for necroptosis-induced tissue damage in vivo.

## Discussion

The knowledge on programmed necrotic cell death has advanced rapidly in recent years. However, the regulatory mechanisms of TNF- and TLRs-induced necroptosis remain unclear. Our study has revealed that JNK differentially regulate TNF- and TLRs-induced necroptosis in macrophages. We discovered that both JNK activities and their scaffold functions were required for TNF-induced necroptosis, while JNK showed kinase-dependent and independent roles in TLRs-induced necroptosis (Fig. 7j). We found that JNK kinase activities promoted TNF-induced SIRS and *Staphylococcus aureus*-mediated lung damage in mice. Other study showed a lethal phenotype of JNK1 and JNK2 double knockout mice^46^, which indicate that the JNK scaffold function within their proteins may serve as a brake for their kinase activities to prevent the uncontrolled necroptosis and tissue damage. Thus, our study determines a dual role for JNK as necroptosis regulators in vitro and in vivo.

We found that LPS or poly I:C can induce RIPK1 and RIPK3 kinases dependent production of pro-inflammatory cytokines under the condition that caspase 8 activity was inhibited as previously reported^36^. The previous paper has reported that LPS plus zVAD induced more ERK activation in BMDMs^36^. However, we found that in peritoneal macrophages, LPS plus zVAD trigger comparable ERK activation and even lower ERK activation at 6h (Fig. 1a). These differences might be due to the different primary cells used and different doses of stimuli. Enhanced activation of JNK and P38 was absent in the macrophages treated with RIPK1 inhibitor Nec-1 and RIPK3 knockdown macrophages. Our results indicated that JNK and P38 were the downstream mediators of RIPK1 or RIPK3 that promoted the induction of pro-inflammatory cytokines. However, the detailed mechanism of how RIPK1 and RIPK3 promote the activation of JNK and P38 is not clear, which needed to be further investigated.

RIPK1 is a critical component for necrosome formation, and its autophosphorylation is indispensable for the activation of necroptosis. By loss of the RIPK1 protein itself, TNF-induced necroptosis is blocked while TLRs-induced necroptosis is promoted. The regulation of JNK in necroptosis is very similar to that of RIPK1. Here, we found that JNK associated with RIPK1 in TNF- and TLRs-triggered necroptosis. Both inhibition of JNK with SP600125 and loss of JNK with siRNA inhibited the autophosphorylation of RIPK1. In addition, JNK knockdown had no effect on necroptosis in RIPK1 loss macrophages triggered with TNF or TLRs. The data suggested that JNK regulated necroptosis dependent on RIPK1. And the autophosphorylation of RIPK1 might be required for JNK-regulated necroptosis. JNK1 and JNK2 double-deficient mice were embryonically lethal^46^. Our results may offer an explanation of lethality of JNK1 and JNK2 double-deficient mice which is possibly due to increased necroptosis. RIPK1 and RIPK3 double knockout may rescue the death of JNK-deficient mice because JNK-regulated necroptosis is dependent on RIPK1 and RIPK3.

TRIF has been reported to mediate TLRs-induced necroptosis by recruiting RIPK1 and RIPK3 via its RHIM domain, which is dispensable for TNF-mediated necroptosis^44^. Our results showed that depletion of JNK in macrophages and fibroblasts increased the abundance of TRIF oligomers, which could recruit more RIPK1 and RIPK3 to induce necroptosis. In addition, oligomerization of TRIF is critical for downstream signaling, which can also be regulated by selective autophagy^45,47^. It has been reported that JNK2 could positively regulate mitophagy (selective autophagy of mitochondria) via its scaffold activity and not its kinase activity^48^. Therefore, we speculated that JNK could promote selective autophagy of TRIF to inhibit TLRs-induced necroptosis via scaffold activity.

## Electronic supplementary material

Supplementary Information
