# Supplementary material for: c-Jun N-terminal kinases differentially regulate TNF- and TLRs-mediated necroptosis through their kinase-dependent and -independent activities
Source: Cell Death Dis. 2018 Nov 15;9(12):1140. doi: 10.1038/s41419-018-1189-2 (PMC6238001; doi:10.1038/s41419-018-1189-2)
Supplement: Supplementary file 1 — Supplementary Information [file 41419_2018_1189_MOESM1_ESM.docx]

**
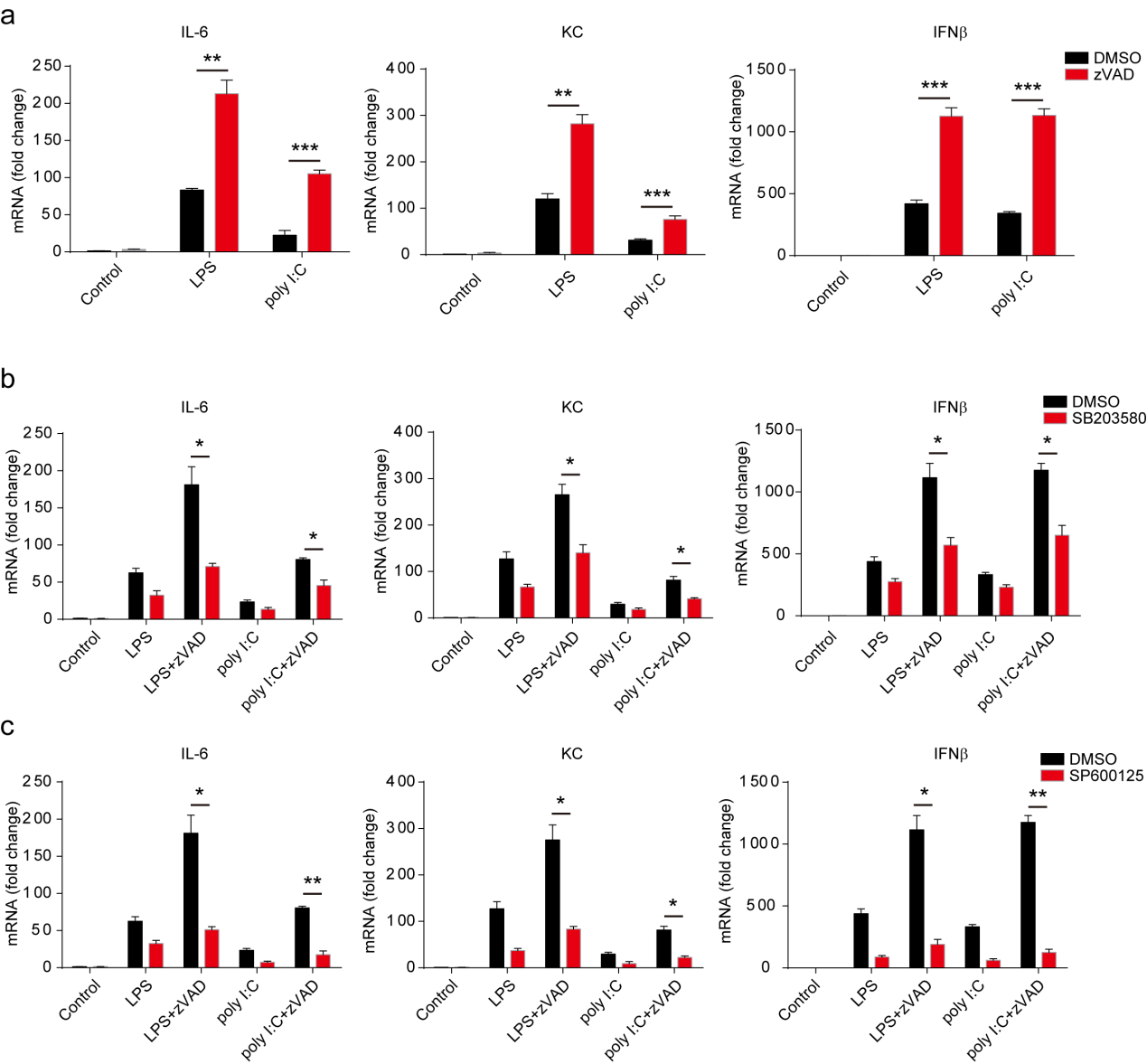
**

**Supplementary Figure 1. P38 and JNK promote RIPK1 and RIPK3-dependent proinflammatory cytokines** **synthesis induced by LPS plus zVAD or poly I:C plus zVAD**.

(**a**) Real time-PCR analysis of *IL-6*, *KC* and *IFNβ* mRNA expression of peritoneal macrophages pretreated with DMSO or zVAD for 30 min, and then untreated or treated with LPS or poly I:C for 3 hours. (**b**) Real time-PCR analysis of *IL-6*, *KC* and *IFNβ* mRNA expression of peritoneal macrophages pretreated with DMSO, SB203580 or zVAD for 30 min and then untreated or treated with LPS or poly I:C for 3 hours. (**c**) Real time-PCR analysis of *IL-6*, *KC* and *IFNβ* mRNA expression in peritoneal macrophages pretreated with DMSO, SP600125 or zVAD for 30 min and then untreated or treated with LPS or poly I:C for 3 hours. Data are representative of at least three independent experiments (mean±SEM). **p* < 0.05, ***p* < 0.01, ****p* < 0.001 by Student’s t test.


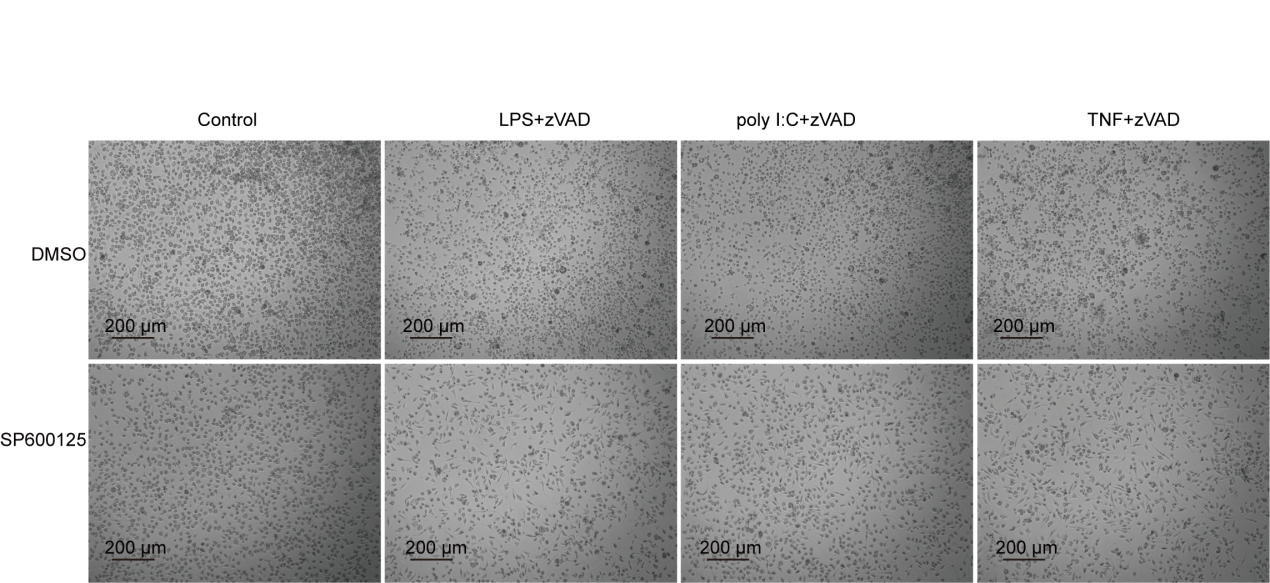


**Supplementary Figure 2 Related to Figure 2. SP600125 suppresses TNF- or TLRs-induced necroptosis.**

Representative images of peritoneal macrophages pretreated with DMSO, SP600125 or zVAD for 30 min and then untreated or treated with LPS, poly I:C and TNF for 12 hours. Photomicrographs of histology are shown at 100× magnification. Data are representative of at least three independent experiments.


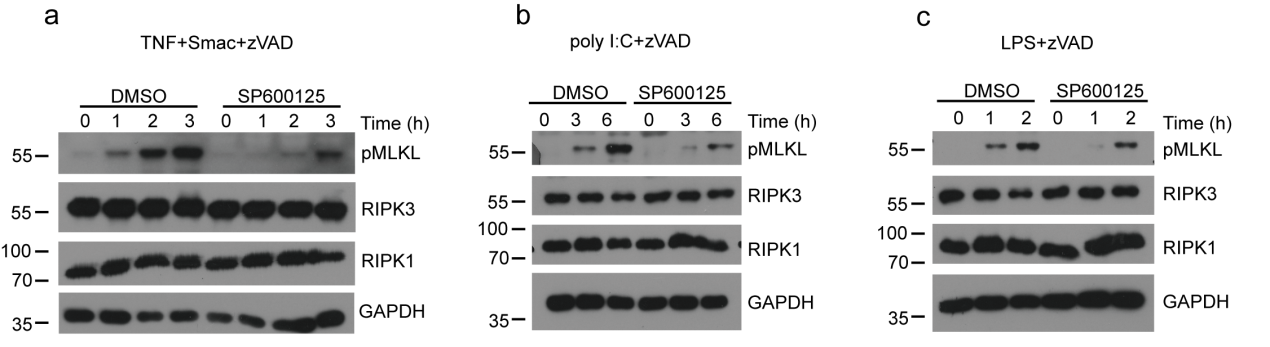


**Supplementary Figure 3 Related to Figure 3. JNK inhibition with SP600125 reduces the phosphorylation of MLKL in Raw 264.7 induced by LPS+zVAD, poly I:C+zVAD or TNF+Smac+zVAD.**

(**a**-**c**) Raw 264.7 cells were pretreated with DMSO, SP600125 or zVAD for 30 min and then treated with LPS (**a**), poly I:C (**b**) and TNF+Smac (**c**) for the indicated time. Cell lysates were analyzed by immunoblotting using the indicated antibodies. Data are representative of at least three independent experiments.


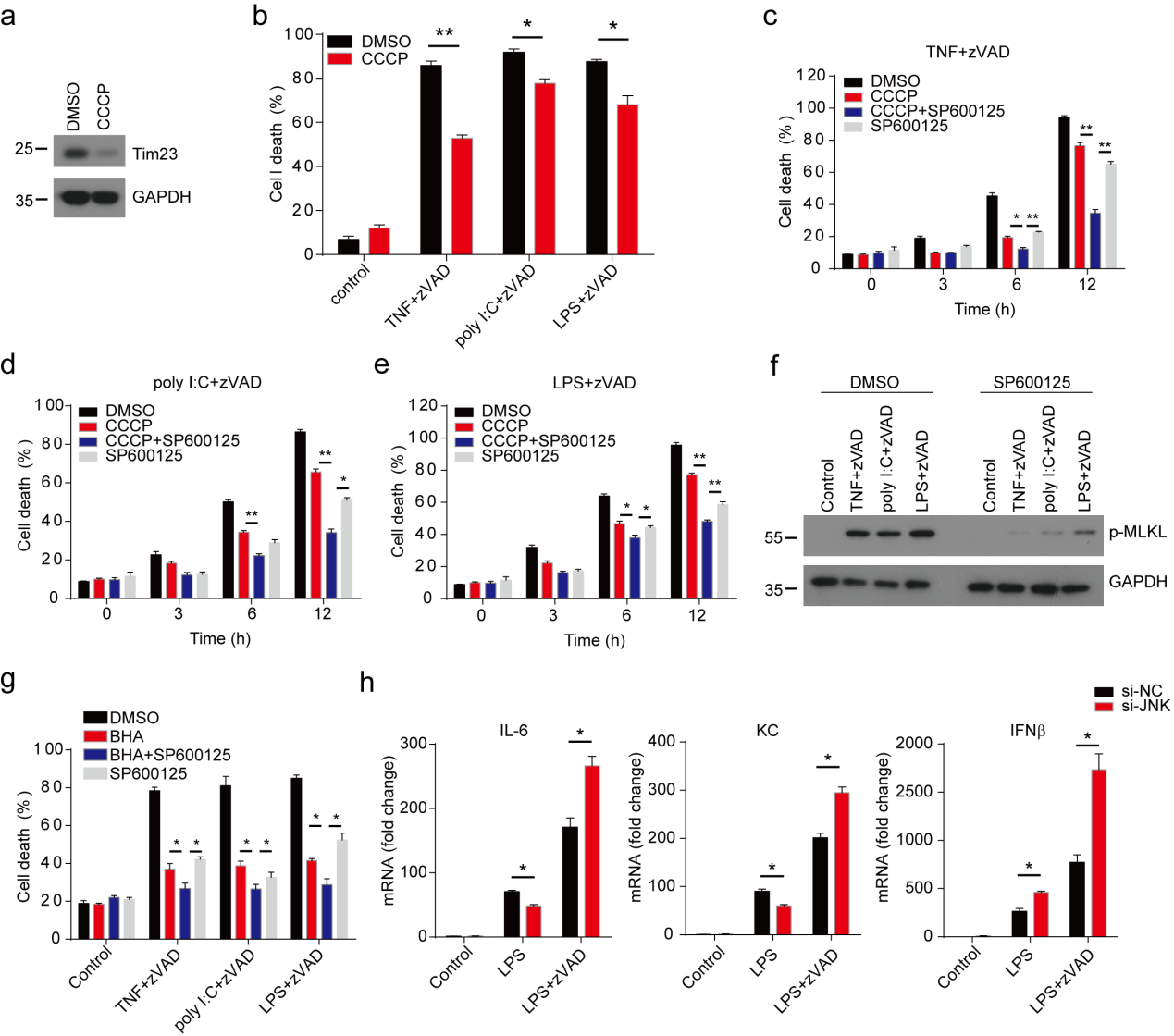


**Supplementary Figure 4. The regulation of necroptosis by JNK is independent of mitochondrial ROS**

(**a**) Immunoblot analysis of peritoneal macrophages treated with protonophore carbonylcyanide m-chlorophenylhydrazone (CCCP) (12.5 μM) for 24 hours. (**b**) Peritoneal macrophages were treated with DMSO or CCCP for 24 hours, followed by the TNF+zVAD, poly I:C+zVAD and LPS+zVAD treatment for additional 12 hours. Cell death was measured by released LDH. (**c**-**e**) Peritoneal macrophages were treated with DMSO or CCCP for 24 hours. Then, peritoneal macrophages were treated with indicated inhibitors and then treated with TNF+zVAD (**c**), poly I:C+zVAD (**d**) and LPS+zVAD (**e**) for the indicated time. Cell death was measured by released LDH. (**f**) Peritoneal macrophages were treated with CCCP for 24 hours and then treated with DMSO, SP600125 or zVAD for 30 min, followed by TNF, poly I:C, LPS for 3 hours. Immunoblot analysis of MLKL phosphorylation in peritoneal macrophages. (**g**) Peritoneal macrophages were pretreated with DMSO, BHA (100μM), zVAD or SP600125 for 30 min, followed by TNF, poly I:C or LPS for 6 hours. Cell death was determined by released LDH. (**h**) Peritoneal macrophages were transfected with si-NC or si-JNK for 3 days and then treated with zVAD for 30 min, followed by LPS for 3 hours. The mRNA level of *IL-6*, *KC* and *IFNβ* were analyzed by real time-PCR.

Data are representative of at least three independent experiments in graphs **b**-**e**, **g** and **h** (mean±SEM). **p* < 0.05, ***p* < 0.01 by Student’s t test.


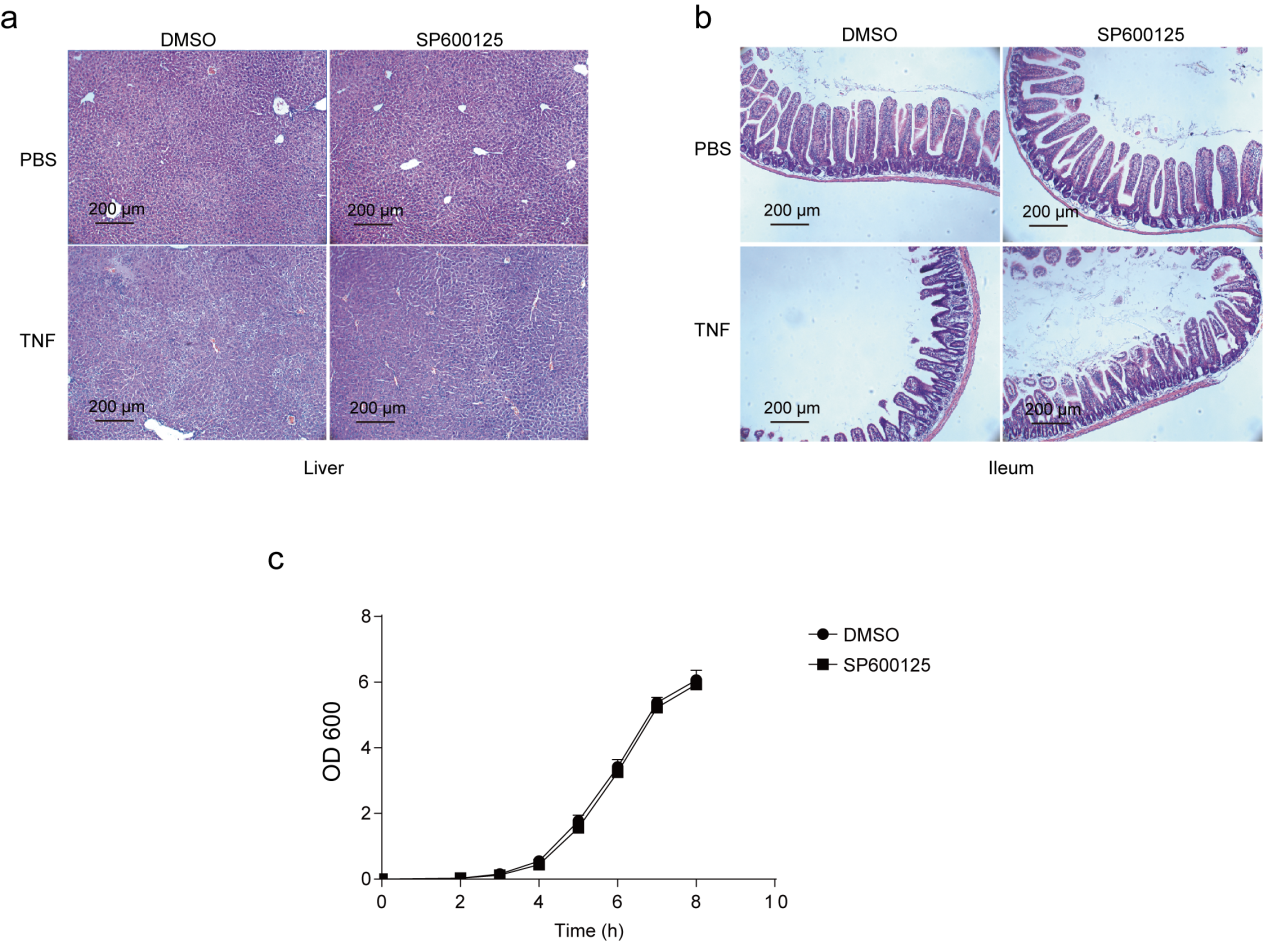


**Supplementary Figure 5 Related to Figure 7. The liver and ileum of mice treated with SP600125 are similar to those of mice treated with DMSO in TNF-induced SIRS model.**

(**a**,**b**) H&E histology of representative liver (**a**) and ileum (**b**) from mice treated with DMSO+PBS, SP600125+PBS, DMSO+TNF or SP600125+TNF for 12 hours in TNF induced SIRS model. (**c**) Growth curve of *Staphylococcus aureus* treated with DMSO or SP600125 *in vitro*.

**Supplementary Table 1.** Real-time PCR Primers related to experimental procedures

| Gene | Direction |  | Primer sequence (5’->3’) |
| --- | --- | --- | --- |
| mouse IL-6 | forward |  | GATGGATGCTACCAAACTGGAT |
|  | reverse |  | CCAGGTAGCTATGGTACTCCAGA |
| mouse KC | forward |  | AGACTCCAGCCACACTCCAA |
|  | reverse |  | TGACAGCGCAGCTCATTG |
| mouse IFNβ | forward |  | CAACAAGTGTCTCCTCCAAAT |
|  | reverse |  | TCTCCTCAGGGATGTCAAAG |
| mouse Rpl13a | forward |  | GGGCAGGTTCTGGTATTGGAT |
|  | reverse |  | GGCTCGGAAGTGGTAGGGG |
| mouse cFLIP | forward |  | TGGACAAAGTGTATGCGTGGAA |
|  | reverse |  | TCACGTAGGAGCCAGGATGAGT |
| mouse A20 | forward |  | TGGGAAGGGACACAACTACA |
|  | reverse |  | GCAGAAACTTCCTCGTCCTC |
| mouse Cyld | forward |  | GGACAGTACATCCAAGACCG |
|  | reverse |  | GAACTGCATGCGGTTGCTC |
| mouse IRAK4 | forward |  | CATACGCAACCTTAATGTGGGG |
|  | reverse |  | GGAACTGATTGTATCTGTCGTCG |
| mouse Myd88 | forward |  | GTCCGACCGTGACGTCCTGC |
|  | reverse |  | CCACCATGCGGCGACACCTT |
| mouse TLR4 | forward |  | ATGGCATGGCTTACACCACC |
|  | reverse |  | GAGGCCAATTTTGTCTCCACA |
| mouse TLR3 | forward |  | GTGAGATACAACGTAGCTGACTG |
|  | reverse |  | TCCTGCATCCAAGATAGCAAGT |
| mouse BIRC2 | forward |  | GTGATGGTGGCTTGAGATGTTG |
|  | reverse |  | CAAGAACTCACACCTTGGAAACC |
| mouse BIRC3 | forward |  | GAAGTGGGCTGCGGTATCA |
|  | reverse |  | GCGCTGTCTTGAACCATGTTC |
| mouse JNK1 | forward |  | TGTGGAATCAAGCACCTTCA |
|  | reverse |  | GCACCCCACAGACCATAAGT |
| mouse JNK2 | forward |  | CCCAAGGAATTGTTTGTGCT |
|  | reverse |  | CGAGTTGACGGTAGGCTCTC |
